# Caffeic Acid Phenethyl Ester Administration Reduces Enterotoxigenic *Bacteroides fragilis*-Induced Colitis and Tumorigenesis

**DOI:** 10.3390/toxins16090403

**Published:** 2024-09-18

**Authors:** Soonjae Hwang, Minjeong Jo, Ju-Eun Hong, Woo-Seung Kim, Da-Hye Kang, Sang-Hyeon Yoo, Kyungsu Kang, Ki-Jong Rhee

**Affiliations:** 1 Department of Biomedical Laboratory Science, College of Software and Digital Healthcare Convergence, Yonsei University MIRAE Campus, Wonju 26493, Republic of Korea; soonjae@gachon.ac.kr (S.H.); jominjeong@skku.edu (M.J.); jehong@yonsei.ac.kr (J.-E.H.); redberry1245@yonsei.ac.kr (W.-S.K.); dahyekang@missouri.edu (D.-H.K.); yshyyb@yonsei.ac.kr (S.-H.Y.); 2 Department of Biochemistry, Lee Gil Ya Cancer and Diabetes Institute, College of Medicine, Gachon University, 155 Gaetbeol-ro, Yeonsu-gu, Inchon 21999, Republic of Korea; 3 Department of Molecular Cell Biology, Sungkyunkwan University School of Medicine, Suwon 16419, Republic of Korea; 4 Department of Obstetrics, Gynecology and Women’s Health, University of Missouri, Columbia, MO 65211, USA; 5 Natural Product Informatics Research Center, Korea Institute of Science and Technology, Gangneung 25451, Republic of Korea; kskang@kist.re.kr

**Keywords:** caffeic acid phenethyl ester, ETBF, BFT, colitis, tumorigenesis

## Abstract

The human colonic commensal enterotoxigenic *Bacteroides fragilis* (ETBF) is associated with chronic colitis and colon cancer. ETBF colonization induces colitis via the *Bacteroides fragilis* toxin (BFT). BFT secreted by ETBF cause colon inflammation via E-cadherin cleavage/NF-κB signaling. ETBF promotes colon tumorigenesis via interleukin 17A (IL-17A)/CXCL-dependent inflammation, but its bioactive therapeutics in ETBF-promoted tumorigenesis remain unexplored. In the current study, we investigated the caffeic acid phenethyl ester (CAPE) in the murine model of ETBF colitis and tumorigenesis. In this study, we observed that CAPE treatment mitigated inflammation induced by ETBF in mice. Additionally, our findings indicate that CAPE treatment offers protective effects against ETBF-enhanced colon tumorigenesis in a mouse model of colitis-associated colon cancer induced by azoxymethane (AOM) and dextran sulfate sodium. Notably, the decrease in colon tumorigenesis following CAPE administration correlates with a reduction in the expression of IL-17A and CXCL1 in the gastrointestinal tract. The molecular mechanism for CAPE-induced protection against ETBF-mediated tumorigenesis is mediated by IL-17A/CXCL1, and by NF-κB activity in intestinal epithelial cells. Our findings indicate that CAPE may serve as a preventive agent against the development of ETBF-induced colitis and colorectal cancer (CRC).

## 1. Introduction

Colorectal cancer (CRC) is on the rise globally, and patients with CRC demonstrate the highest mortality rates among the major types of cancer [1,2,3]. A crucial tumor-promoting factor is chronic inflammation in the large intestine [4]. This observation is particularly evident in patients with inflammatory bowel disease (IBD), who show a markedly elevated incidence of colorectal cancer (CRC) [4,5,6,7]. Among the various environmental factors that may contribute to intestinal inflammation, the intestinal microbiota has been implicated to be a crucial factor [8]. Thus, extensive efforts have been performed to identify bacterial species that promote colitis and/or colonic tumorigenesis as well as elucidate the mechanism(s) by which these microbes modulate the inflammatory response [9].

Enterotoxigenic *Bacteroides fragilis* (ETBF) is a pathogenic human commensal bacterium that induces colonic inflammation in mice [10,11,12,13,14,15,16]. ETBF strains secrete the *Bacteroides fragilis* toxin (BFT) exotoxin that damages the epithelial junctions, resulting in subsequent inflammatory immune response in large intestinal tissues [10,11]. As the prevalence of ETBF is greater in colorectal cancer (CRC) patients than in healthy individuals [17,18,19,20], understanding the mechanism by which BFTs promote tumorigenesis is an area of intense research. In animal models of ETBF-induced tumorigenesis, the BFT was shown to exacerbate colitis in tumorigenesis by promoting the production of the inflammatory cytokine interleukin-17A (IL-17A) and CXCL1 [21,22,23] in the host. The BFT is a zinc-dependent metalloprotease that induces cleavage of E-cadherin via γ-secretase-dependent signal transduction [24]. E-cadherin cleavage compromises intestinal integrity and triggers NF-κB signaling in colon epithelial cells [25,26,27,28]. In addition, BFT exposure induces CXCL1 expression and secretion in colon epithelial cells [11,26].

Currently, the available options for the clearance of ETBF infection are limited. Treatment with cefoxitin, an antibiotic, eliminates ETBF in mice, thereby diminishing inflammatory response in the large intestine of the host [29]. However, the non-specific targeting of the cefoxitin may induce the survival of cefoxitin-resistant microbes in the gut, which can lead to the overgrowth of *Clostridium difficile* in the gut. A high prevalence of toxigenic *C. difficile* strains is clinically associated with the development of CRC [9]. Therefore, the utilization of natural products and nutritional agents is gaining attention for their potential in treating ETBF-mediated colitis and tumorigenesis. Previously, an in vivo study by our group showed that zerumbone suppressed ETBF-induced colitis in mice and BFT-induced NF-κB signaling in human colonic epithelial cells via inhibiting NF-κB signaling [30] and colonic tumorigenesis [31]. However, few effective therapies can specifically target ETBF-infection-induced colitis and tumorigenesis.

Caffeic acid phenethyl ester (CAPE) is a polyphenolic chemical of natural products that possesses numerous biological activities, including anti-inflammatory effects [32,33,34,35,36,37,38,39,40,41]. CAPE directly suppresses transcriptional NF-κB activity [42,43,44]. However, to the best of our knowledge, there are no studies that investigated the beneficial effect of CAPE in ETBF-mediated diseases. Consequently, this study was conducted to examine the effects of CAPE in a murine model of ETBF-mediated colitis and tumorigenesis, as well as to investigate the underlying mechanisms in vitro.

## 2. Results

### 2.1. CAPE Administration Decreases ETBF-Induced Colitis

**Figure 1:**
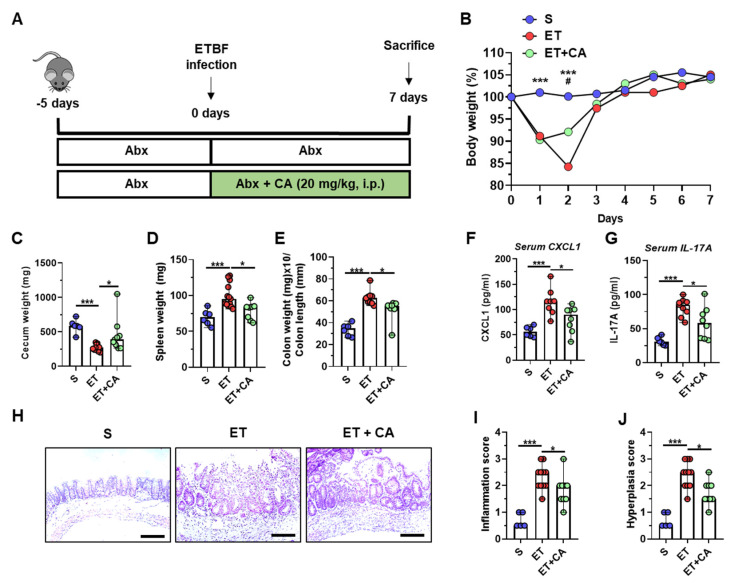
CAPE treatment protects mice against ETBF-induced colitis. C57BL/6 female mice were given water ad libitum supplemented with clindamycin and gentamicin for 5 days prior to WT-ETBF (1 × 10^9^ CFU) infection. Following WT-ETBF infection, the antibiotic cocktail was administered continuously for an additional 7 days. During WT-ETBF infection, mice were administered daily with CAPE (20 mg/kg, i.p.). Mice were sacrificed and all parameters were assessed at day 7 post-infection. (**A**) Experimental design. (**B**) Body weight. The daily body weight of each mouse was expressed as a percentage of the initial body weight. ET vs. S, *** *p* < 0.001; ET+CA vs. ET, ^#^ *p* < 0.05; significances between treated groups were determined using Mann–Whitney U test. (**C**) Cecum weight (mg). (**D**) Spleen weight (mg). (**E**) Colon weight (mg)/colon length (mm). (**F**) Serum CXCL1 levels. (**G**) Serum IL-17A levels. (**H**) Histology (H&E) of the cecum, ×200 magnification; scale bar, 100 μm. (**I**) Inflammation scores. (**J**) Hyperplasia scores. S, sham; ET, WT-ETBF; CA, caffeic acid phenethyl ester. Each dot corresponds to a single mouse (*n* = 4−12 mice per group) in the bar plots. Horizontal bar, median. * *p* < 0.05, *** *p* < 0.001.

Previous studies have shown that CAPE diminished the progression of colitis in a T-cell transfer model or dextran sulfate sodium (DSS) model of colitis [45,46]; however, the role of CAPE in ETBF-induced colitis has not been yet determined. It has been shown that the severity of ETBF-induced colitis correlates with decreased body weight, decreased cecum weight, increased spleen weight, and increased ratio of colon weight/colon length in mice [10,47]. CAPE was administered to mice as follows (Figure 1A). The results showed that the weight loss in ETBF-colonized mice was significantly higher than that of the CAPE-treated ETBF group on day 2; however, by day 3, the levels of weight loss were similar to those of the ETBF-infected group treated with CAPE (Figure 1B). CAPE treatment did not affect ETBF colonization (Supplementary Figure S1). Cecum weight analysis revealed that ETBF-infected mice given CAPE showed less loss of cecum weight (Figure 1C). In addition, treatment with CAPE resulted in a reduction in the spleen weight and colon weight-to-length ratio when compared to ETBF-infected mice (Figure 1C,D).

To analyze more direct inflammation parameters, mouse sera were examined for inflammatory cytokines. The inflammatory cytokines, CXCL1 and IL-17A, were reduced in the sera of ETBF-colonized mice treated with CAPE compared to mice infected with ETBF alone (Figure 1F,G). Histopathologic analysis indicated that ETBF-infected mice given CAPE showed less inflammation and hyperplasia on day 7 (Figure 1H–J) compared to ETBF-infected mice. These findings suggest that the administration of CAPE offers protection against the onset of inflammation in a mouse model of ETBF-induced colitis.

### 2.2. CAPE Administration Protects against ETBF-Induced Colonic Tumorigenesis

Consistent with the increased risk of CRC observed in patients infected with ETBF [18], recent studies showed that ETBF infection promoted colonic tumor development in ETBF-induced colitis and tumorigenesis models [21,22]. Therefore, the ETBF-mediated tumorigenesis model was utilized to assess the impact of CAPE treatment on the development of ETBF-induced AOM/DSS tumorigenesis.

**Figure 2:**
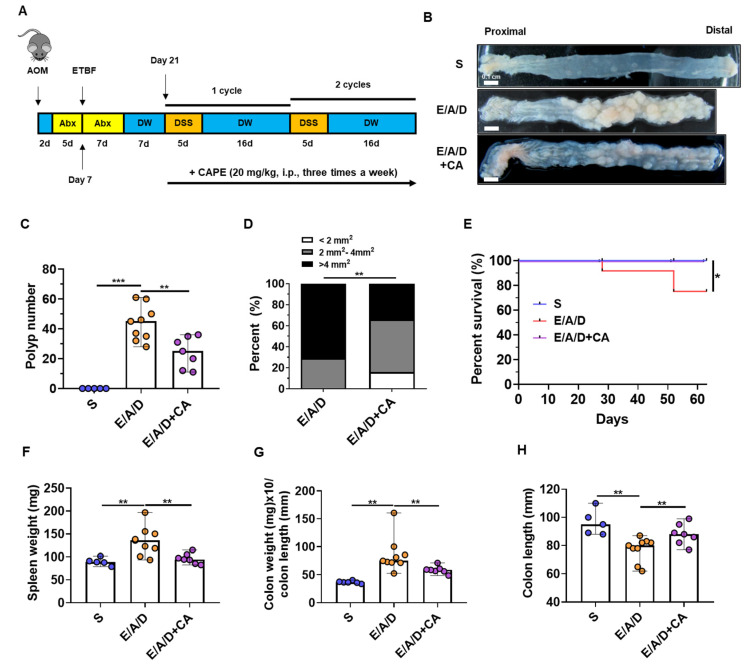
CAPE treatment protects mice against ETBF-promoted development of colonic tumorigenesis. C57BL/6 mice received a single intraperitoneal injection of AOM (10 mg/kg) and were provided with ad libitum access to drinking water containing clindamycin and gentamicin for 5 days (yellow box). Following this, ETBF was orally administered, and the antibiotic regimen continued for an additional 7 days (yellow box). Seven days later, the mice underwent two cycles of 1% DSS treatment (five days each cycle, orange boxes) followed by distilled water (DW; sixteen days each cycle, blue boxes). Throughout the two DSS cycles, C57BL/6 mice were administered CAPE (20 mg/kg, intraperitoneally, three times a week). The entire duration of the experiment spanned 9 weeks. (**A**) Schematic overview of ETBF-promoted azoxymethane (AOM)/dextran sulfate sodium (DSS)-induced tumorigenesis model, and CAPE treatment. (**B**) Representative gross macroscopic image of the colon. (**C**) Polyp number per mouse. (**D**) Polyp size distribution. (**E**) Survival curve of C57BL/6 mice. Kaplan–Meier curves depicting survival after CAPE treatment in ETBF/AOM/DSS model, Mantel–Cox log-rank test. (**F**) Spleen weight (mg). (**G**) Colon weight (mg)/colon length (mm). (**H**) Colon length (mm). In the bar plots, each dot corresponds to a single mouse. The horizontal bar denotes the median. S, sham control; E, ETBF; A, AOM; D, DSS; CA, CAPE. Results were pooled from three independent experiments (*n* = 5–9 mice per group). * *p* < 0.05; ** *p* < 0.01; *** *p* < 0.001.

AOM/DSS mice infected with ETBF received CAPE (20 mg/kg) during the two DSS cycles, administered three times a week as described in Materials and Methods (Figure 2A). CAPE treatment did not influence ETBF colonization (Supplementary Figure S2). Mice were euthanized after the second DSS cycle. It was observed that the number and size of tumors in the CAPE-treated ETBF+AOM/DSS group were significantly lower and smaller, respectively, than those in ETBF+AOM/DSS mice (Figure 2B–D). Furthermore, the ETBF-infected AOM/DSS mice given CAPE exhibited reduced morbidity compared to the ETBF+AOM/DSS group (Figure 2E). In line with the observed reduction in tumorigenesis and mortality, the AOM/DSS mice infected with ETBF and treated with CAPE displayed decreased spleen weight, a lower colon weight-to-length ratio, and increased colon length (Figure 2F–H). These findings suggest that CAPE offers protection against ETBF-induced colon tumorigenesis associated with AOM/DSS treatment.

### 2.3. CAPE Treatment Attenuated Microadenoma Progression in ETBF-Mediated Tumorigenesis Model

**Figure 3:**
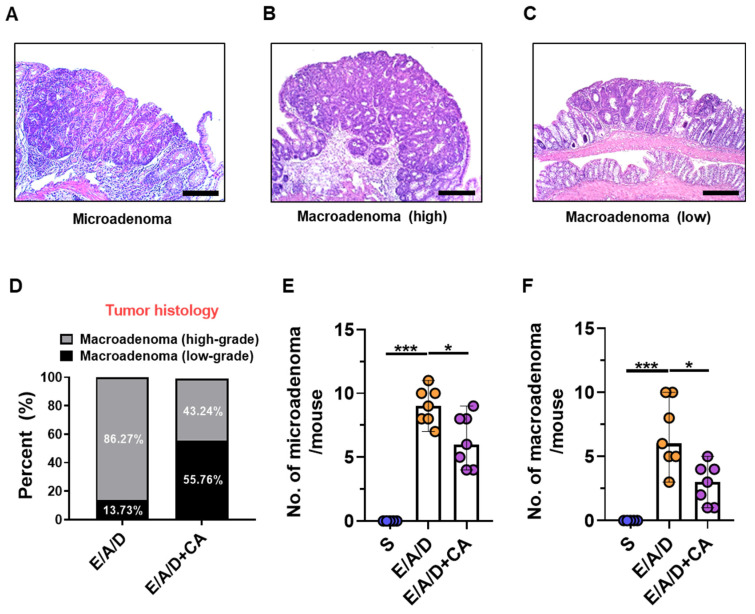
CAPE treatment decreased tumor progression in ETBF-mediated tumorigenesis model. C57BL/6 mice treated with AOM were infected with ETBF and underwent two cycles of 1% DSS for 9 weeks. Throughout these two DSS cycles, the mice received CAPE (20 mg/kg, intraperitoneally, three times a week). (**A**) Representative images of microadenomas after ETBF colonization in AOM/DSS-treated mice, ×100 magnification; scale bar, 100 μm. (**B**) Representative images of high-grade macroadenomas after ETBF colonization in AOM/DSS-treated mice, ×100 magnification; scale bar, 100 μm. (**C**) Representative images of low-grade macroadenomas after CAPE treatment in ETBF-colonized AOM/DSS mice, ×50 magnification; scale bar, 200 μm. (**D**) Macroadenoma distribution of low grade and high grade. (**E**) Number of microadenomas per mouse. (**F**) Number of macroadenoma per mouse. Bar plot. Horizontal bar, median. S, sham control; E, ETBF; A, AOM; D, DSS; CA, CAPE. * *p* < 0.05, *** *p* < 0.001. The significance between the treated groups was assessed using the Mann–Whitney U test.

Tumorigenesis of the large intestine involves a sequence of changes, beginning from a healthy mucous membrane, then the occurrence of excessive epithelial cell proliferation and formation of aberrant crypt foci (ACF), and finally leading to the formation of microadenoma to macroadenoma through malignant transformation [48,49]. Macroadenoma is an adenoma developed from microadenoma that is not grossly visible and indicates nuclear atypia and loss of nuclear polarity [50]. CAPE treatment resulted in a reduction in the number of polyps formed in the ETBF-infected AOM/DSS group. To determine at which stage of tumorigenesis CAPE exerted its protective effects, we quantified microadenomas, low-grade macroadenomas, and high-grade macroadenomas (Figure 3A–C). Histological staining indicated that the proportion of high-grade macroadenomas was lower in the ETBF+AOM/DSS group treated with CAPE compared to the control ETBF/AOM/DSS group (Figure 3D). Similarly, the numbers of microadenomas and macroadenomas were significantly reduced in the CAPE-treated ETBF+AOM/DSS group relative to the control group (Figure 3E,F). These findings suggest that CAPE primarily inhibits the initiation and promotion of adenomas in the ETBF-mediated tumorigenesis model.

### 2.4. CAPE-Mediated Reduction in Colitis from ETBF-Mediated Tumorigenesis Model

**Figure 4:**
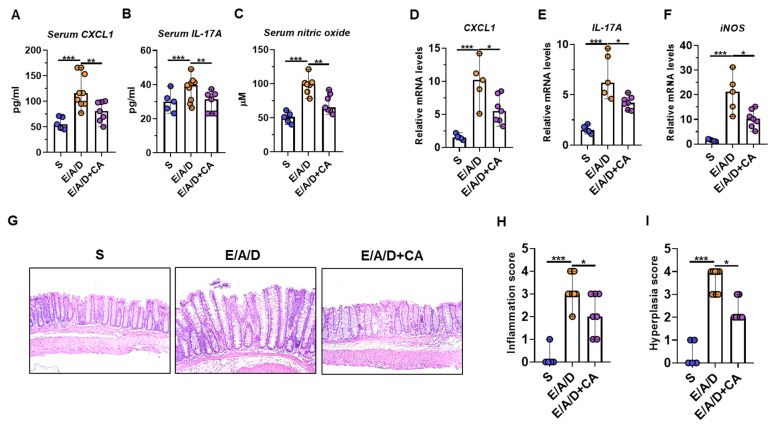
CAPE reduces the expression of pro-inflammatory genes in ETBF-colonized tumorigenesis mice. Distal colon and serum were analyzed for the mRNA expression of CXCL1, IL-17A, and iNOS by qRT-PCR and ELISA, separately. Relative values of qRT-PCR were normalized to those of *gapdh*. Serum nitrite level was examined by nitric oxide assay. (**A**) Serum CXCL1 levels. (**B**) Serum IL-17A levels. (**C**) Serum nitric oxide levels. (**D**) CXCL1 expression. (**E**) IL-17A expression. (**F**) iNOS expression. (**G**) Histology (H&E) of distal colon tissues, 100× magnification; scale bar, 100 μm. (**H**) Inflammation score. (**I**) Hyperplasia score. Each dot represents one mouse. Bar plot. Horizontal bar, median. S, sham control; E, ETBF; A, AOM; D, DSS; CA, CAPE. * *p* < 0.05; ** *p* < 0.01; *** *p* < 0.001. Significance between treated groups was determined using Mann–Whitney U test.

ETBF infection promotes tumorigenesis via IL-17A/CXCL1 inflammatory cytokines [21,22]. Furthermore, the expression of inducible nitric oxide synthase (iNOS) induced by inflammatory cytokines was commonly noted during the progression of colon cancer [51]. Given that CAPE treatment diminished several indicators of colonic inflammation in the ETBF-induced colitis model (Figure 2), we further validated the anti-inflammatory effects of CAPE in the ETBF tumorigenesis model. Serum levels of CXCL1, IL-17A, and nitric oxide were lower in the CAPE-treated ETBF/AOM/DSS group compared to the ETBF/AOM/DSS group (Figure 4A–C). In line with the serum data, the expression of pro-inflammatory cytokine genes (CXCL1, IL-17A, and iNOS) was also reduced in the colonic tissues of CAPE-treated ETBF/AOM/DSS mice compared to the control ETBF/AOM/DSS mice (Figure 4D–F). Histologic analysis confirmed decreased inflammation in the non-tumor-bearing colon tissues of CAPE-treated ETBF/AOM/DSS mice compared to the control ETBF/AOM/DSS mice (Figure 4H,I). In addition, hyperplasia scores from CAPE-treated ETBF/AOM/DSS mice were significantly lower than those in the control ETBF/AOM/DSS mice (Figure 4J). Collectively, these findings suggest that CAPE inhibits tumor promotion in ETBF-mediated tumorigenesis by reducing inflammation.

### 2.5. CAPE Reduced BFT-Induced IL-8 Expression and NF-κB Activity in Colon Epithelial Cells

**Figure 5:**
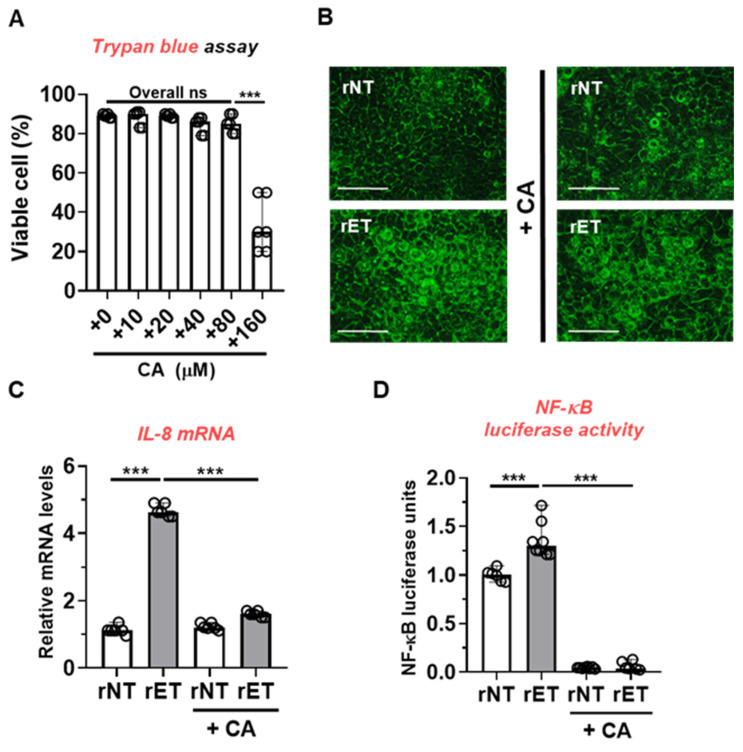
CAPE suppresses BFT-induced IL-8 expression and NF-κB luciferase activity in intestinal epithelial cells. HT29/C1 cells were exposed to CAPE either alone or in combination with rETBF (rET) culture supernatants (1:10). (**A**) The cell viability of HT29/C1 cells treated with CAPE (ranging from 10 to 160 μM) was assessed after 24 h, both with and without rETBF (rET) culture supernatants (1:10). (**B**) Changes in cell morphology were observed. HT29/C1 cells were treated either with rETBF (rET) supernatant (positive control) alone or with rETBF (rET) supernatant in conjunction with 80 μM CAPE. rNTBF (rNT) supernatant served as a negative control. The morphological changes of the cells were examined using microscopy. Magnification, ×400. Scale bar, 100 μm. (**C**) qRT-PCR analysis of IL-8 expression in HT29/C1 cells treated with rET culture supernatant and 80 μM CAPE for 3 h. (**D**) NF-κB luciferase activity of BFT incubated HT29/C1 cells treated with 80 μM CAPE for 3 h. Luciferase activity was normalized to *Renilla* luciferase activity, and the relative values were reported. C, control; CA, caffeic acid phenethyl ester. *** *p* < 0.001; ns, no statistical significance. Significance between treated groups was determined using Mann–Whitney U test.

Our data clearly showed that treatment with CAPE led to protection against ETBF-induced colitis and colitis-associated tumorigenesis. To investigate the anti-inflammatory mechanism of CAPE in ETBF colitis-promoted tumorigenesis, we employed the human colonic carcinoma cell line HT29/C1 to examine BFT-induced inflammatory signaling. Colonic epithelial cells treated with BFTs undergo cell rounding, which is dependent on E-cadherin cleavage. This response leads to the activation of the NF-κB pathway to promote *Cxcl1* expression in the colonic epithelial cell line [27]. Initially, to identify the highest concentration of CAPE that does not cause cytotoxicity, HT29/C1 cells were treated with various concentrations of CAPE (ranging from 10 to 160 μM) for 24 h, and cell viability was assessed using the trypan blue exclusion assay. The results showed that cell viability in HT29/C1 cells decreased at a concentration of 160 μM CAPE (Figure 5A). Consequently, we selected 80 μM of CAPE to examine its effects on BFT-induced cell rounding, IL-8 (the human equivalent of mouse CXCL1) expression, and NF-κB luciferase activity.

As active BFT exposure induces cellular rounding via E-cadherin cleavage in HT29/C1 cells, HT29/C1 cells were co-treated with the culture supernatant of recombinant B. fragilis secreting the active BFT (rET) and 80 μM CAPE for 24 h, after which cellular rounding was analyzed using microscopy. As expected, rNT-treated cells did not undergo cell rounding irrespective of CAPE treatment (Figure 5B). However, rET-treated cells cotreated with CAPE underwent cell rounding similarly to cells treated with rET alone. In support of this, CAPE did not prevent E-cadherin cleavage in BFT-treated cells (Supplementary Figure S3). To gain further mechanistic insights, IL-8 expression and NF-κB luciferase activity were measured in HT29/C1 cells. Analysis of qPCR results revealed a reduction in IL-8 expression in HT29/C1 cells co-treated with rET and CAPE (80 μM) compared to those treated with rET alone (Figure 5C). Additionally, assessment of NF-κB luciferase activity demonstrated a decrease in NF-κB activity in rET-exposed HT29/C1 cells treated with CAPE compared to the control group treated solely with rET (Figure 5D). These findings indicate that CAPE treatment mitigates BFT-induced CXCL1/NF-κB signaling in colonic epithelial cells.

## 3. Discussion

With the global rise in colorectal cancer (CRC) risk, considerable efforts have been made to comprehend and address the pathogenesis of CRC. Numerous clinical studies and laboratory models have established that a human commensal, ETBF, plays a crucial role in aggravating chronic inflammation in the colon and contributing to CRC development [23,47,52,53,54]. In the present study, we are the first to demonstrate the anti-tumorigenesis effects of CAPE on ETBF-associated CRC. Our findings suggest that CAPE inhibits ETBF-induced tumorigenesis by blocking BFT-mediated inflammatory CXCL1/NF-κB signaling. Further, these findings suggest that CAPE is involved in a distinct preventive mechanism against BFT-mediated colon inflammation compared to the conventional modulation of inflammatory signaling in DSS [46] or a TNBS colitis model [45].

Inflammatory cytokines and histological scores were reduced in ETBF-infected mice treated with CAPE (20 mg/kg) compared to control mice infected solely with ETBF. BFTs target epithelial adherence junctions [55] and induce the ectodomain cleavage of E-cadherin, thereby destroying the epithelial barrier and activating the NF-κB/β-catenin pathway [27,28]. We verified the inhibitory effects of CAPE on BFT-induced CXCL1/NF-κB signaling in intestinal epithelial cells. While the molecular mechanisms underlying this CAPE-mediated suppression of tumorigenesis may vary based on the experimental context, the primary common function of CAPE appears to be the reduction in BFT-induced inflammation. Our data indicate that CAPE treatment decreases BFT-induced IL-8 (the human equivalent of CXCL1) expression by inhibiting NF-κB in intestinal epithelial cells, resulting in reduced inflammation in the large intestine during colitis and CRC.

The ETBF-infection-induced NF-κB pathway in colonic epithelial cells has been reported to be activated by IL-17A cytokines secreted by Th17 or γδT cells [21]. Since CAPE is an NF-κB inhibitor, the secretion of IL-17A cytokines by Th17 or γδT cells may be inhibited by CAPE. According to one report, in the experimental autoimmune encephalomyelitis (EAE) model in which the Th17 immune response is pathogenic, pre-treatment with CAPE inhibited the translocation of NF-κB in T cells and further increased the expression of Foxp3 in T cells, which promoted the differentiation of regulatory T cells in tissues, leading to the inhibition of EAE development [56]. Additionally, activation of p-STAT3 is essential for naïve T cells to be differentiated into Th17 cells [57]. Since CAPE has been reported as a STAT3 inhibitor, it will be effective in suppressing Th17 differentiation. Activation of the STAT3 pathway has been documented in the ETBF-induced tumorigenesis model.

The strong hydrophobicity and chemical instability of CAPE result in low bioavailability, significantly limiting its application in functional foods [58]. Due to the unstable properties of CAPE, research has been conducted on enhancing its stability and increasing its delivery efficiency to intestinal mucosal tissues using nanotechnology [59]. The method of delivering CAPE through synthetic nanoparticle carriers may inadvertently lead to mucosal toxicity depending on the concentration and properties of the carrier. CAPE is a polyphenolic active ingredient found in propolis, which naturally contains high levels of this compound, particularly in New Zealand propolis [60,61,62,63,64]. Oral consumption of New Zealand propolis can enhance the stability of CAPE and improve its bioavailability. Additionally, propolis contains various bioactive substances with anti-inflammatory and anticancer properties. Therefore, further research is needed to investigate the efficacy of propolis containing CAPE in the context of inflammation and tumorigenesis induced by ETBF infection.

In this study, our preliminary findings suggest that CAPE treatment did not prevent BFT-induced cleavage of full-length E-cadherin (Supplementary Figure S3) and cell rounding. However, it remains to be seen whether CAPE affects the sequential degradation of E-cadherin induced by BFT. It has been shown previously that treatment with CAPE exhibited neuroprotective effects via modulating α-secretase and β-secretase activity in the hippocampal cell culture system [65]. CAPE-mediated suppression of CXCL1/NF-κB may be partly via γ-secretase blockade, which prompted us to make a preliminary hypothesis that CAPE plays the role of γ-secretase inhibitor. However, there were no structural similarities between CAPE and several γ-secretase inhibitors containing L-685,458 (Supplementary Figure S4), which was analyzed by ChemMine tool, an online-based software, to provide an analysis program comparing the structure between two chemicals.

The Sears group showed that the γ-secretase inhibitor suppressed the BFT-induced nuclear location of β-catenin, thereby suppressing cellular proliferation in HT29/C1 cells [24]. Although nuclear β-catenin was not examined in the current study, as CAPE suppressed NF-κB signaling and induced intracellular E-cadherin fragments, CAPE may be superior to zerumbone, which was previously investigated in the ETBF-mediated colitis [30] and CRC model [31]. Further research might be required to evaluate CAPE’s potential efficacy compared with zerumbone in ETBF-induced colon inflammation and the development of CRC.

Although our findings highlight the protective role of CAPE in safeguarding against ETBF-induced colitis and tumor development, it is crucial to explore its wider effects on the intestinal mucosa and the composition of gut microbiota. Research data suggest that, when applying CAPE to DSS-mediated IBD, it was found that CAPE produced significant protective effects against enteritis [59,66]. This protection was associated with enhancements in the gut microbiome and shifts in metabolite profiles, indicating that CAPE not only eases inflammation but also fosters a more favorable microbial community in the gut. In addition, investigating how CAPE influences the integrity of the intestinal barrier, particularly through its interactions with epithelial cells, could provide valuable insights into its role in preserving gut health and preventing dysbiosis.

In this study, we showed that CAPE treatment reduced ETBF-mediated colitis by downregulating the expression of IL-17A and CXCL1 in mice. Additionally, we discovered that CAPE inhibited tumorigenesis promoted by ETBF in an azoxymethane (AOM)/dextran sulfate sodium (DSS)-induced animal model. Our findings suggest that CAPE can be a pharmacological candidate for ETBF-mediated colitis and tumorigenesis and provide additional insight into the function of CAPE in ameliorating ETBF-induced CRC.

## 4. Conclusions

We found a protective effect of CAPE in ETBF-induced colitis and colonic tumorigenesis in a murine model. Potential mechanisms underlying the anti-tumorigenic effects of CAPE involve the reduction in inflammation induced by ETBF infection and the inhibition of the BFT-mediated CXCL1/NF-κB pathway in colonic epithelial cells. These data suggest that CAPE may be used as a supplementary therapy to augment existing treatments in ETBF infections.

## 5. Materials and Methods

### 5.1. Animals

Female C57BL/6 mice (18–20 g; 8 weeks) were obtained from Raon-Bio Co. (Yongin, Republic of Korea) and maintained under specific pathogen-free conditions. Mice were maintained at 25 °C with a 12 h light/dark phase cycle. All procedures were reviewed and followed according to the Institutional Animal Care and Use Committee (IACUC) guidelines at the Yonsei University Mirae campus at Wonju. All experimental procedures for animal experiments have been examined and passed by the IACUC of Yonsei University MIRAE campus (YWCI-201612-014-01, YWCI-201901-002-01) and the Institutional Biosafety Committee (IBC) of Yonsei University MIRAE campus (201612-P-014-01, 201809-P-005-01). According to the findings from the Sears group [21], there were no differences in inflammation and pathology between female and male mice induced by ETBF. Additionally, females were selected for the experiment due to their lower incidence of inter-individual aggression and greater ease of handling compared to males. Although we have not confirmed the anti-inflammatory and anti-cancer effects of CAPE in male mice, we anticipate that these protective effects will also be evident in male mice infected with ETBF.

### 5.2. Human Model Intestinal Epithelial Cells

The human colon epithelial cell line HT29/C1 (kindly gifted by Cynthia Sears, Johns Hopkins University) was maintained in Dulbecco′s modified Eagle medium (DMEM, 4.5 g/L glucose, L-glutamine) supplemented with 1.5 mM L-glutamine and 2.2 g/L sodium bicarbonate, 10% (*v*/*v*) heat-inactivated fetal bovine serum (FBS), 200 IU/mL penicillin, and 200 µg/mL streptomycin at 37 °C in a humidified incubator with 5% CO_2_. Culture media were replaced 2 times per week, and the cell line was trypsinized with 0.25% trypsin-EDTA solution (Sigma, St. Louis, MO, USA) following standard procedures.

### 5.3. Cell Stimulation with Bacteroides fragilis Toxin

Culture supernatants of *B. fragilis* were sterilized using a syringe filter (0.45 μm, Merck, Rahway, NJ, USA) to remove bacteria, as previously described [18], and stored at −80 °C. HT29/C1 cells were treated with the bacterial culture supernatants from recombinant strains of *B. fragilis*, specifically NCTC9343 (rETBF) (pFD340::P-bft, which secretes wild-type BFT-2) or NCTC9343 (rNTBF) (pFD340::P-bftΔH352Y, which secretes a mutated, biologically inactive form of BFT). The culture media of HT29/C1 cells were washed with PBS and subsequently replaced with serum-free media to minimize the neutralization of BFT by serum proteins. The supernatants from each recombinant strain of *B. fragilis* were diluted to a 1:10 ratio in serum-free media. HT29/C1 cells were treated with caffeic acid phenethyl ester (CAPE) (Sigma, St. Louis, MO, USA), BAY 11-7085 (an NF-κB inhibitor; Calbiochem, San Diego, CA, USA), and L-685,458 (a γ-secretase inhibitor; Sigma, St. Louis, MO, USA) in conjunction with the culture supernatants of the *B. fragilis* recombinant strains. All culture media for bacteria and human colon cancer cell lines, as well as chemical reagents, were sourced from GIBCO Life Technologies (Rockville, MD, USA) unless otherwise indicated.

### 5.4. Treatment with CAPE in ETBF-Mediated Colitis Model

Female C57BL/6 mice were given access to water and standard chow ad libitum. All mice were housed for a minimum of 7 days before initiation of experimental protocols (day 0). Mice have been orally inoculated with 200 μL of *B. fragilis* strains via an inoculation needle. The wild-type enterotoxigenic *B. fragilis* (ETBF) strain secreting BFT-2 (B. fragilis 86-5443-2-2) was utilized to infect C57BL/6 mice, resulting in colitis. All strains of *B. fragilis* employed in this study are resistant to clindamycin and gentamicin, and they were generously provided by Cynthia Sears and Augusto Franco-Mora from Johns Hopkins University (Baltimore, MD, USA). Female C57BL/6 mice received an antibiotic cocktail (clindamycin 0.1 g/L and gentamicin 3 g/L) in their drinking water. The ETBF or ETBF+CAPE groups were subsequently administered approximately 10^8^ CFU of ETBF in PBS via oral injection. For preparing bacterial cultures for oral administration, 50 mL of *B. fragilis* culture media was centrifuged (11,000× *g*, 25 °C, for 30 min). The resulting pellets of *B. fragilis* were then re-suspended in 1.0 mL of PBS to achieve a concentration of 10^8^ CFU/mL. The CFU in each inoculum was assessed through serial dilution plating on BHIB agar. Fecal colonization by *B. fragilis* was evaluated by vortexing fresh fecal pellets in 1 mL of PBS, followed by serial dilution plating on BHIB agar [10]. The BFT-producing wild-type *B. fragilis* strain or the biologically inactive mutant was cultured anaerobically in a BHI medium (composed of 37 g of brain heart infusion base [Difco Laboratories, Detroit, MI, USA] per liter, 5 g of yeast extract [Difco Laboratories, Detroit, MI, USA] per liter, 5 μg of hemin/mL, and 0.5 g of L-cysteine/mL) for 24 h. Throughout the experimental period, all groups were given antibiotics in their drinking water. The two groups included ETBF-infected mice receiving either the vehicle (200 μL/20 g/day i.p.) or CAPE (20 mg/kg/day i.p.) daily for 7 days, in accordance with the experimental design (Figure 1A). To investigate the anti-inflammatory effects of CAPE, mice were administered CAPE by intraperitoneal injection every day after oral inoculation of ETBF. After ETBF infection, body weight was examined daily until the mice were sacrificed (Figure 1A). Control groups were administered the vehicle (PBS) daily for 7 days as appropriate. Treatment with CAPE commenced simultaneously with the oral injection of ETBF in the mice.

### 5.5. CAPE Treatment in ETBF-Promoted AOM/DSS-Induced Tumorigenesis

To evaluate the anti-tumorigenic effects of CAPE on ETBF-mediated tumorigenesis, mice intraperitoneally received a single injection of AOM (10 mg/kg) and were given distilled water containing antibiotics (clindamycin and gentamicin) two days later for a total of 12 days (Figure 2A). ETBF was orally administered once on day 7. On day 21, the first cycle of DSS treatment (5 days of 1% DSS followed by 16 days of distilled water) was initiated, comprising a total of two DSS cycles. Intraperitoneal administration of CAPE began one week after ETBF colonization and continued until the conclusion of the experiment on day 63 (Figure 2A). Throughout both DSS cycles, mice were treated with CAPE intraperitoneally three times a week at a dosage of 20 mg/kg, in accordance with the experimental design (Figure 2A). On day 63, the colons of mice from all groups were extracted and cut longitudinally. The number and size of tumors in both the proximal and distal sections of the colons were assessed, and approximately 2 cm of tissue was frozen for RNA analysis. The colons were immediately fixed in 10% formalin and processed for histopathological examination.

### 5.6. Tumor Enumeration and Histopathology

Mice were sacrificed for histological analysis, and then a portion of the tumor-bearing tissue was fixed in 10% formalin. Immediately before immersing the tissue in formalin, the size of the polyp was measured and recorded by classifying it as follows: polyp size was calculated as length × width, with polyps categorized into three groups: <2 mm^2^, 2–4 mm^2^, and >4 mm^2^. The results are presented as the median polyp size. To enable a longitudinal examination of the entire colon, the colons were “Swiss-rolled” before embedding and sectioning. For histological analysis under a microscope, formalin was removed from the fixed tissue during the tissue processing step, followed by the preparation of a paraffin block. Tissues were embedded in paraffin and sectioned to 6 μm using a microtome. Histological slides were stained with hematoxylin, bluing buffer, and eosin for 1 min each, dehydrated with alcohol (95%, 100%, 100%) for 2 min each, and then rinsed in xylene twice for 1 min each. The H&E-stained slides were evaluated for aberrant crypt foci, microadenomas, low-grade macroadenomas, and high-grade macroadenomas by two independent investigators. All slides were quantified in representative fields, with 10 randomly selected fields at 200× magnification per specimen. Representative images were captured using an optical microscope and processed using Adobe Photoshop 7.0 (Adobe, San Jose, CA, USA).

### 5.7. Quantification of Human Cytokine Expressions

RNA was extracted from HT29/C1 cells using TRI_ZOL_ extraction reagent (Invitrogen, Burlington, ON, Canada), and cDNA was synthesized using the high-capacity cDNA synthesis kit (Invitrogen, Carlsbad, CA, USA) with 4000 ng of template RNA. qPCR was performed using primers against GAPDH (Thermo Fisher Scientific, Carlsbad, CA, USA) and CXCL1 (Thermo Fisher Scientific, Carlsbad, CA, USA), and normalized against the reference genes GAPDH, as previously described [30].

### 5.8. Trypan Blue Exclusion Assay

HT29/C1 cells were plated in 24-well culture plates and incubated with varying concentrations of CAPE or the NF-κB inhibitor (BAY 11-7082) at the specified doses. After 24 h, the culture supernatants were removed and washed with PBS, after which the cells were harvested using 0.25% trypsin/EDTA (Life Technologies, Carlsbad, CA, USA) for 5 min at 37 °C. The detached HT29/C1 cells were then mixed with DMEM supplemented with 10% FBS to neutralize the trypsin and inhibit its enzymatic activity. The percentage of viable cells was determined using Trypan blue dye (0.4%), as previously described [30].

### 5.9. ELISA

Hemolysis-free sera were obtained from ETBF-colonized C57BL/6 mice given CAPE through cardiac puncture. After coagulation at 4 °C, each mouse’s blood was centrifuged (12,000× *g*, 4 °C, 20 min). The supernatants of blood were transferred to the autoclaved microfuge tube. Sera were stored at −80 °C until measured. Mouse CXCL1 and IL-17A were quantified in hemolysis-free sera using ELISA kits (R&D system, Minneapolis, MN, USA) according to the manufacturer’s instructions.

### 5.10. Histologic Assessment of Inflammation and Hyperplasia

The large intestine of mice was excised and then washed with cold PBS to eliminate fecal contents. Following the sacrifice of the mouse, the cecum and colon were excised, and the luminal contents were rinsed out using cold PBS. The wet weight of the organs was subsequently measured. Likewise, the excised spleen was placed directly onto a petri dish and weighed immediately. The cecum and colon free of fecal contents were fixed with 10% formalin (Sigma) for 7 days at 4 °C. After fixation of 7 days, the fixed tissues were performed in the tissue processor (Leica) to replace the penetrated formalin in tissues with ethanol. The ethanol was also replaced with xylene for the end of tissue processing. The xylene-penetrated tissues were incubated with molten paraffin to make formalin-fixed embedded (FFPE) tissues. The FFPE blocks were sectioned to make slides coated with tissue sections (4 μm) for hematoxylin and eosin (H&E) and visualized using an image analysis software LAS 2.0 (Leica, Wetzlar, Germany). Histological scoring was based on assessments of crypt damage, regeneration, inflammation, and injury. The final inflammation score was calculated as the sum of scores across all parameters. Inflammation was classified as follows: 0 for normal conditions; 1 for mild immune cell infiltration without alterations to the colon epithelium; 2 for moderate immune cell infiltration accompanied by mild colon epithelial proliferation; and 3 for severe immune cell infiltration with abnormal colon epithelial proliferation and significant loss of crypt architecture. Hyperplasia was graded as follows: 0 for normal; 1 for a mild increase in crypt length; 2 for moderate crypt length accompanied by hyperchromatic colon epithelial cells; and 3 for a severe increase in crypt length with a high mitotic index. The tissue slides were scored through a blinded evaluation by a certified biomedical laboratory scientist specialized in mouse tissue pathology. Representative images were captured using an optical microscope and processed with Adobe Photoshop.

### 5.11. Luciferase Reporter Assay

HT29/C1 cells were plated in 24-well plates and incubated for 24 h before transfection. Reporter constructs, including NF-κB luciferase and Renilla luciferase plasmids, were transfected using Lipofectamine^®^ 3000 (Invitrogen, Carlsbad, CA, USA). After 48 h of transfection, the cells were treated with BFT supernatant, CAPE (R&D Systems, Minneapolis, MN, USA), and L-685,458 (R&D Systems, Minneapolis, MN, USA) for an additional 24 h. The luciferase activity in HT29/C1 cells was assessed using the Dual-Luciferase^®^ reporter assay system (Promega, Madison, WI, USA). Measurements of luciferase activity were conducted using a GloMax^®^ 20/20 luminometer (Promega, Madison, WI, USA), with firefly luciferase activity normalized to Renilla luciferase activity.

### 5.12. Structural Similarity Analysis 

The structural similarity assessment between CAPE and γ-secretase inhibitors was conducted using ChemMine tools (https://chemminetools.ucr.edu/ (accessed on 6 September 2024). ChemMine tools is an online service designed for the analysis of small-molecule data. It offers a web interface that includes a collection of cheminformatics and data mining tools, which are beneficial for various analytical processes in chemical genomics and drug discovery. Pairwise structural similarities among compounds were quantified as previously described [67]. A Tanimoto value of 0.4 or higher, whether measured by AP or MCS, suggests a significant degree of structural similarity between the assessed compounds [67]. The results of the structural similarity analysis between the two compounds calculated using the ChemMine tool are presented in Supplementary Figure S4.

### 5.13. Data Analysis

Statistical significance was tested by GraphPad Prism 8 (La Jolla, San Diego, CA, USA). Results were considered to be statistically significant if *p* < 0.05. Results were presented as the median values across experimental groups. Comparisons between the two groups were conducted using the nonparametric two-tailed Mann–Whitney U test.

## Data Availability

The data utilized in the current study are available upon request from the corresponding author.

## Supplementary Materials

The following supporting information can be downloaded at: https://www.mdpi.com/article/10.3390/toxins16090403/s1. Figure S1. CAPE does not impact ETBF colonization in mice. ETBF colonization was assessed by stool plating; Figure S2. CAPE does not impact ETBF colonization in ETBF-colonized AOM/DSS mice; Figure S3. CAPE does not inhibit the BFT-induced degradation of full-length E-cadherin; Figure S4. Similarity Workbench analysis applied to ChemMine tools.
