# Supplementary material for: Caffeic Acid Phenethyl Ester Administration Reduces Enterotoxigenic Bacteroides fragilis-Induced Colitis and Tumorigenesis
Source: Toxins (Basel). 2024 Sep 18;16(9):403. doi: 10.3390/toxins16090403 (PMC11435740; doi:10.3390/toxins16090403)
Supplement: Supplementary file 1 [file toxins-16-00403-s001.zip › toxins-3156089-supplementary.pdf]

[Supplementary data]

Caffeic Acid Phenethyl Ester Administration Reduces Enterotoxigenic *Bacteroides fragilis*-induced Colitis and Tumorigenesis

Figure S1. CAPE does not impact ETBF colonization in mice. ETBF colonization was assessed by stool plating.

Figure S2. CAPE does not impact ETBF colonization in ETBF-colonized AOM/DSS mice.

Figure S3. CAPE does not inhibit the BFT-induced degradation of full-length E-cadherin.

Figure S4. Similarity Workbench analysis applied to ChemMine tools.

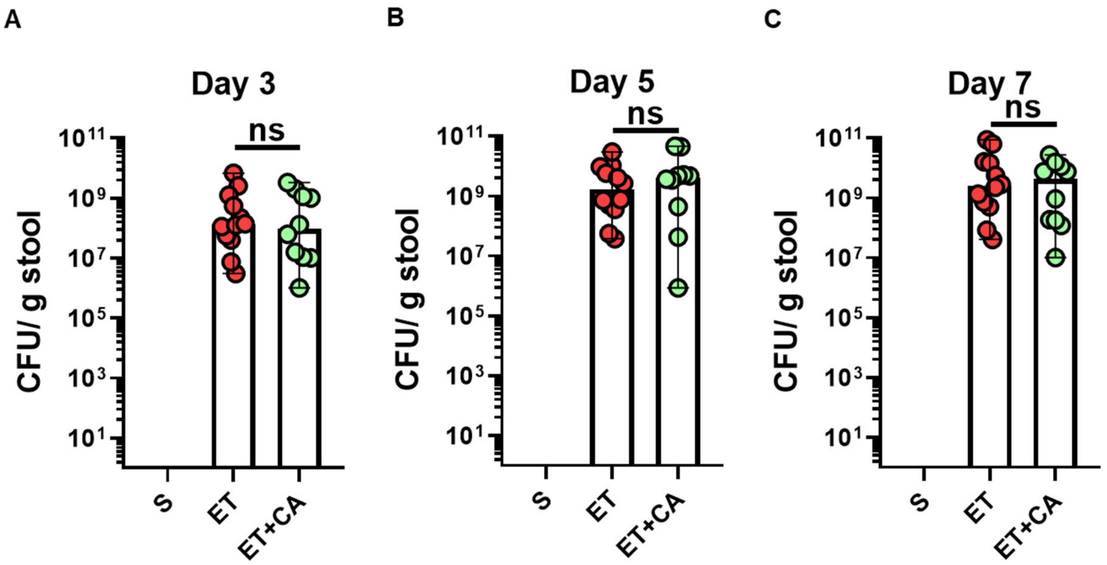

Figure S1. CAPE does not impact ETBF colonization in mice. ETBF colonization was assessed by stool plating. C57BL/6 female mice were inoculated with ETBF (~1 × 10^9 CFU). Colonization was generally within the range of 10^7–10^9 CFU/g stool at day 3 (A), day 5 (B), and day 7 (C) after ETBF infection. Data are expressed as the mean ± SEM from three independent experiments. S, sham; CA, caffeic acid phenethyl ester; ET, ETBF; CFU, colony-forming units; nd, not detected. In the bar plots, each dot corresponds to a single mouse. Significance between treated groups was determined using Mann–Whitney *U* test. ns, no statistical significance.

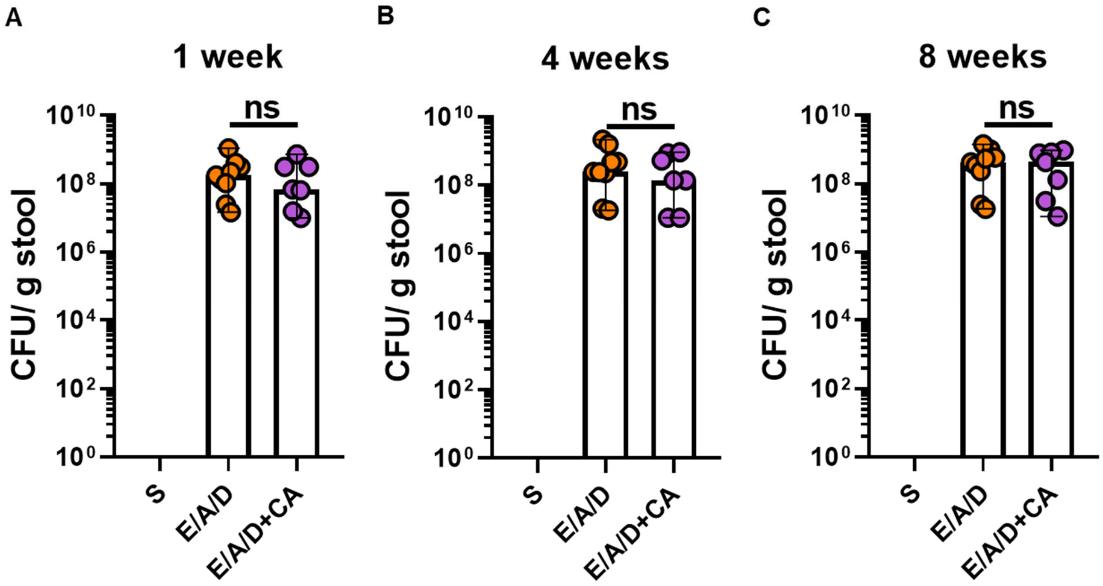

Figure S2. CAPE does not impact ETBF colonization in ETBF-colonized AOM/DSS mice. WT-ETBF colonization was assessed by stool plating. C57BL/6 female mice were inoculated with WT-ETBF (~1×10^9 CFU). Colonization was generally within the range of 10^7–10^9 CFU/g stool at 1 week (A), 4 weeks (B), and 8 weeks (C) after ETBF infection. Data are expressed as the mean ± SEM from three independent experiments. In the bar plots, each dot corresponds to a single mouse. S, sham control; E, ETBF; A, AOM; D, DSS; CA, CAPE; CFU, colony-forming units; nd, not detected. Significance between treated groups was determined using Mann–Whitney *U* test. ns, no statistical significance.

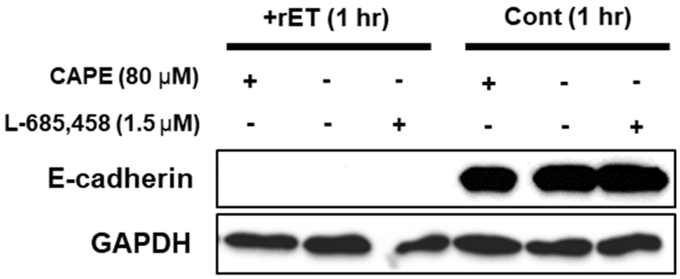

Figure S3. CAPE does not inhibit BFT-induced degradation of full-length E-cadherin in intestinal epithelial cells. HT29/C1 cells were treated with CAPE (80 μM) or the γ-secretase inhibitor L-685,458 (1.5 μM) along with rETBF (rET) culture supernatants (1:10) or fresh BHIB media (control group; Cont) for indicated times. Protein levels of E-cadherin and GAPDH were assessed by Western blotting. The experiment was conducted to determine whether the γ-secretase inhibitor or CAPE can prevent the degradation of full-length E-cadherin induced by BFT. The results indicate that CAPE does not inhibit the degradation of full-length E-cadherin induced by BFT.

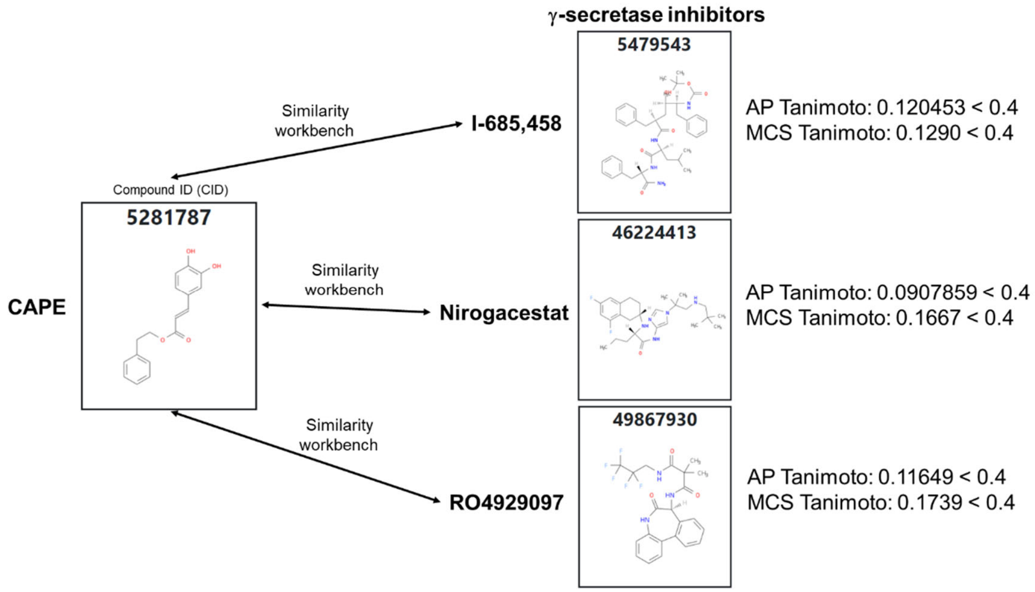

Figure S4. Similarity Workbench analysis applied to ChemMine tools. Structural similarity analysis between CAPE and γ-secretase inhibitors (L-685,485, Nirogacestat and RO4929097) using the ChemMine tool(https://chemminetools.ucr.edu/), an online service for analyzing and clustering small molecules. an AP or MCS Tanimoto value of 0.4 or higher is considered indicative of significant structural similarity between the evaluated compounds. The three γ-secretase inhibitors were confirmed to be structurally dissimilar to CAPE using the ChemMine tool.
